# *Usp18* Driven Enforced Viral Replication in Dendritic Cells Contributes to Break of Immunological Tolerance in Autoimmune Diabetes

**DOI:** 10.1371/journal.ppat.1003650

**Published:** 2013-10-24

**Authors:** Nadine Honke, Namir Shaabani, Dong-Er Zhang, George Iliakis, Haifeng C. Xu, Dieter Häussinger, Mike Recher, Max Löhning, Philipp A. Lang, Karl S. Lang

**Affiliations:** 1 Institute of Immunology, Faculty of Medicine, University of Duisburg-Essen, Essen, Germany; 2 Department of Gastroenterology, Hepatology and Infectious Diseases, Heinrich-Heine-University Düsseldorf, Düsseldorf, Germany; 3 Department of Pathology, Division of Biological Sciences and Moores UCSD Cancer Center, University of California, San Diego, La Jolla, California, United States of America; 4 Institute of Medical Radiation Biology, Faculty of Medicine, University of Duisburg-Essen, Essen, Germany; 5 Clinic for Primary Immunodeficiencies, Medical Outpatient Unit, and Immunobiology, Department of Biomedicine, University Hospital Basel, Basel, Switzerland; 6 Department of Rheumatology and Clinical Immunology, Charité – University Medicine Berlin and German Rheumatism Research Center (DRFZ), Berlin, Germany; La Jolla Institute for Allergy and Immunology, United States of America

## Abstract

Infection with viruses carrying cross-reactive antigens is associated with break of immunological tolerance and induction of autoimmune disease. Dendritic cells play an important role in this process. However, it remains unclear why autoimmune-tolerance is broken during virus infection, but usually not during exposure to non-replicating cross-reactive antigens. Here we show that antigen derived from replicating virus but not from non-replicating sources undergoes a multiplication process in dendritic cells in spleen and lymph nodes. This enforced viral replication was dependent on *Usp18* and was essential for expansion of autoreactive CD8^+^ T cells. Preventing enforced virus replication by depletion of CD11c^+^ cells, genetically deleting *Usp18*, or pharmacologically inhibiting of viral replication blunted the expansion of autoreactive CD8^+^ T cells and prevented autoimmune diabetes. In conclusion, *Usp18*-driven enforced viral replication in dendritic cells can break immunological tolerance and critically influences induction of autoimmunity.

## Author Summary

Autoimmune diabetes in humans is linked to infection with viruses, which carry cross-reactive antigens. Virus derived cross-reactive antigens break immunological tolerance to pancreatic islets, which initiates disease. Several other non-viral sources of cross-reactive antigens are known, however they usually fail to induce diabetes. Here we found that viral antigen underwent an *Usp18* dependent replication in dendritic cells. This mechanism was essential to generate sufficient amounts of cross-reactive antigen and to expand autoreactive CD8^+^ T cells. Blocking of virus replication by either depletion of dendritic cells, genetic depletion of *Usp18* or pharmacological inhibition of replication blunted expansion of autoreactive CD8^+^ T cells and prevented diabetes. In conclusion we found that enforced virus replication broke the tolerance to self-antigen, which partially explains the strong association of autoimmune diseases with virus infections.

## Introduction

Autoimmune diabetes in humans is characterized by immunological destruction of beta islet cells in the pancreas; this cellular destruction leads to hyperglycemia [1]. T cells specific for beta islet cell antigens play an important role in the development of the disease and have been found to arise after exposure to viruses that contain cross-reactive epitopes [2]–[4]. Viruses known to contain cross-reactive epitopes are enterovirus, rubella virus, and rotavirus. Infection with these viruses is often found during the onset of diabetes [5]–[7]. Recent evidence of the ability of viruses to induce diabetes comes from epidemiological and genetic analyses, which have shown that functional polymorphisms in interferon-regulating genes are strongly associated with autoimmune diabetes [8]–[10]. Thus, viral infection is associated with the onset of autoimmune diabetes in humans, and molecular mimicry is an obvious explanation for the immunological destruction of pancreatic beta cells. Besides viruses, several other pathogens and environmental proteins, such as bovine serum albumin (BSA) and beta-casein, carry cross-reactive epitopes to beta islet cells [11]–[13]. Because both substances are found in cow milk, many people are exposed to those antigens. However, this exposure is not strongly linked to the induction of autoreactive T cells or to the occurrence of autoimmune diabetes [14], [15]. Several bacterial species (e.g. *Escherichia coli*, *Pseudomonas* species, and *Campylobacter*) are known to carry epitopes that are cross-reactive to beta islet cells [16], [17]. Although infection with these opportunistic pathogens will lead to presentation of cross-reactive beta islet antigens in combination with high amounts of bacterial Toll-like receptor (TLR) ligands, the contribution of these bacteria to the incidence of diabetes remains uncertain [18]. Thus, cross-reactive viruses, are more efficient than other cross-reactive antigens in breaking immunologic tolerance.

During the onset of autoimmune diabetes, antigen presenting cells (APCs) in secondary lymphoid organs (SLO) are key players in regulating immunological tolerance and immune activation [1]. With their ability to express costimulatory molecules, APCs like dendritic cells (DCs) or macrophages efficiently prime antigen-specific CD8^+^ T cells [1], [19]. DCs express costimulatory molecules after antigen uptake in combination with pattern recognition receptor ligation. Therefore activation of pattern recognition receptors by pathogen-derived patterns is an important mechanism by which DCs can differentiate between self-antigen and foreign antigen. In addition to costimulatory molecules, the amount of antigen presented is important in determining whether tolerance induction or immune activation will occur [20], [21]. A low amount of presented antigen on DCs induces immunological tolerance against this antigen, even if it is presented in parallel with costimulatory signals [22], [23]. In contrast, DCs loaded with high amounts of antigen may induce immune activation even in the absence of costimulatory molecule expression [22], [24], [25]. Thus, the amount of presented antigen is another independent factor that determines tolerance induction or immune activation. We recently reported that CD169^+^ macrophages enforce virus replication, which enhances adaptive immune response [26]. If dendritic cells participate in antigen amplification and how this affects immunological tolerance remains unknown. To examine the importance of enforced viral replication in the context of autoimmune diabetes, we studied the induction of autoimmune diabetes in the RIP-GP mouse model [27]. In this model the glycoprotein (GP) of lymphocytic choriomeningitis virus (LCMV) is expressed as a transgene under the control of the rat insulin promoter (RIP). Following LCMV infection, LCMV-GP specific CD8^+^ T cells are primed and destroy the LCMV-GP expressing insulin producing pancreatic islet cells leading to autoimmune diabetes.

## Results

### Depletion of dendritic cells blunted early virus replication and prevented autoimmune diabetes

**Figure 1:**
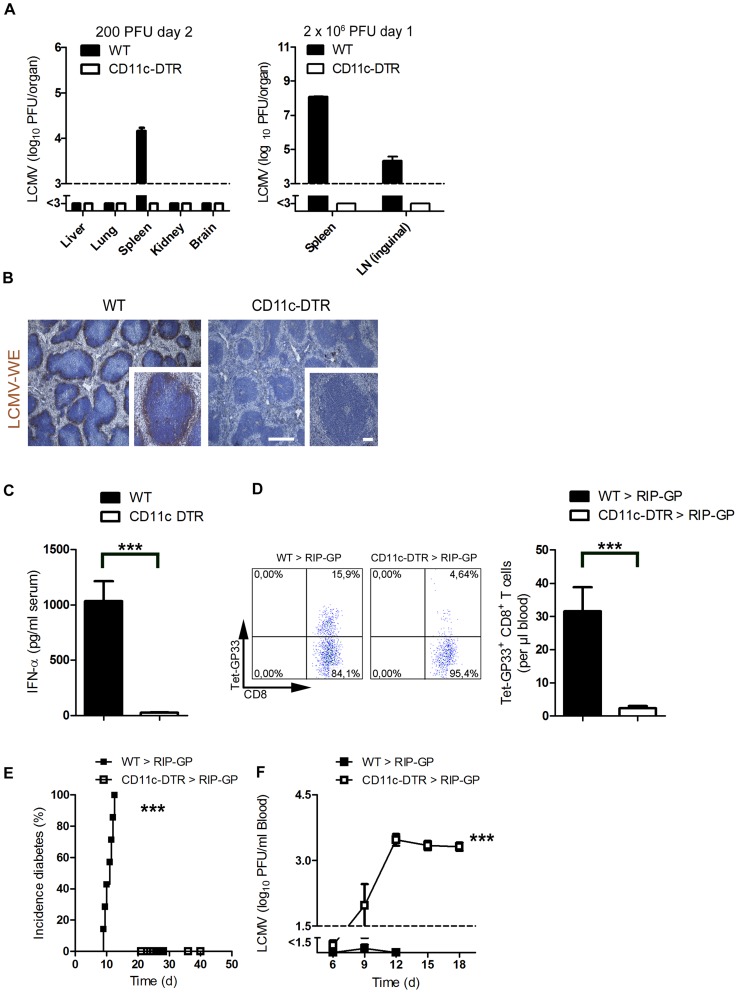
Depletion of dendritic cells blunted early viral replication and prevented autoimmune diabetes. (**A**) CD11c-DTR mice and C57BL/6 mice were treated intraperitoneally with diphtheria toxin (30 µg/kg) on day −3. Mice were infected with LCMV (200 PFU) or LCMV (2×10^6^ PFU) on day 0. Viral titers were analyzed at the indicated time points in different organs (200 PFU n = 6 and 2×10^6^ PFU, n = 3). (**B**) C57BL/6 and CD11c-DTR mice were treated intraperitoneally with diphtheria toxin (30 µg/kg) on day −3 and then infected with LCMV (2×10^6^ PFU) on day 0. After one day, immunohistologic staining for LCMV-NP was performed on spleen sections (n = 3, scale bar main images 500 µm, inlets 100 µm). (**C**) CD11c-DTR mice and control WT mice were treated with 30 µg/kg diphtheria toxin on day −3. On day 0 mice were infected with 2×10^6^ PFU LCMV. After two days IFN-α was measured in the serum by ELISA (n = 6). (D–F) RIP-GP mice were lethally irradiated and one day later were reconstituted with 10^7^ bone marrow cells from either CD11c-DTR mice or C57BL/6 mice as control animals. Thirty days later, mice were treated intraperitoneally with diphtheria toxin (10 µg/kg) on days −1, 2, 5, and 8 and were infected intravenously with 200 PFU LCMV-WE on day 0. A representative dot plot and the quantification of virus specific GP33^+^ CD8^+^ T cells analyzed on day 8 in the blood with FACS analysis is shown (n = 10–14, **D**). The incidence of diabetes was determined by measuring serum glucose concentrations after LCMV infection (n = 7–11, **E**). Virus titers were analyzed in the blood at different time points after infection by plaque assay (n = 5–11, F). *** *P*<0.001 (Student's *t*-test) (C and D), Log-rank (Mantel-Cox) (**E**) two-way analysis of variance (ANOVA) (**F**).

To analyze the contribution of dendritic cells in LCMV replication, we used CD11c-DTR mice. Treatment of CD11c-DTR mice with diphtheria toxin depletes dendritic cells [28]. Lack of dendritic cells completely blunted early LCMV replication in spleen and lymph nodes (Figure 1A). Reduced LCMV replication in the CD11c^+^ compartment impaired viral antigen expressed within the spleen as assessed by immune-histology (Figure 1B). This reduction of replicating antigen in the spleen correlated with the lack of induced interferon-alpha (Figure 1C). Subsequently, the LCMV-specific CD8^+^ T cell response against LCMV-GP was blunted in the absence of dendritic cells (Figure 1D) and CD8^+^ T cell-mediated autoimmune diabetes was prevented (Figure 1E). In the absence of DCs virus could not be controlled and persisted in the blood (Figure 1F). This secondary virus propagation was most likely due to the lack of innate and adaptive immune response [29], [30].

### Pharmacologic reduction of viral replication inhibits onset of autoimmune diabetes

**Figure 2:**
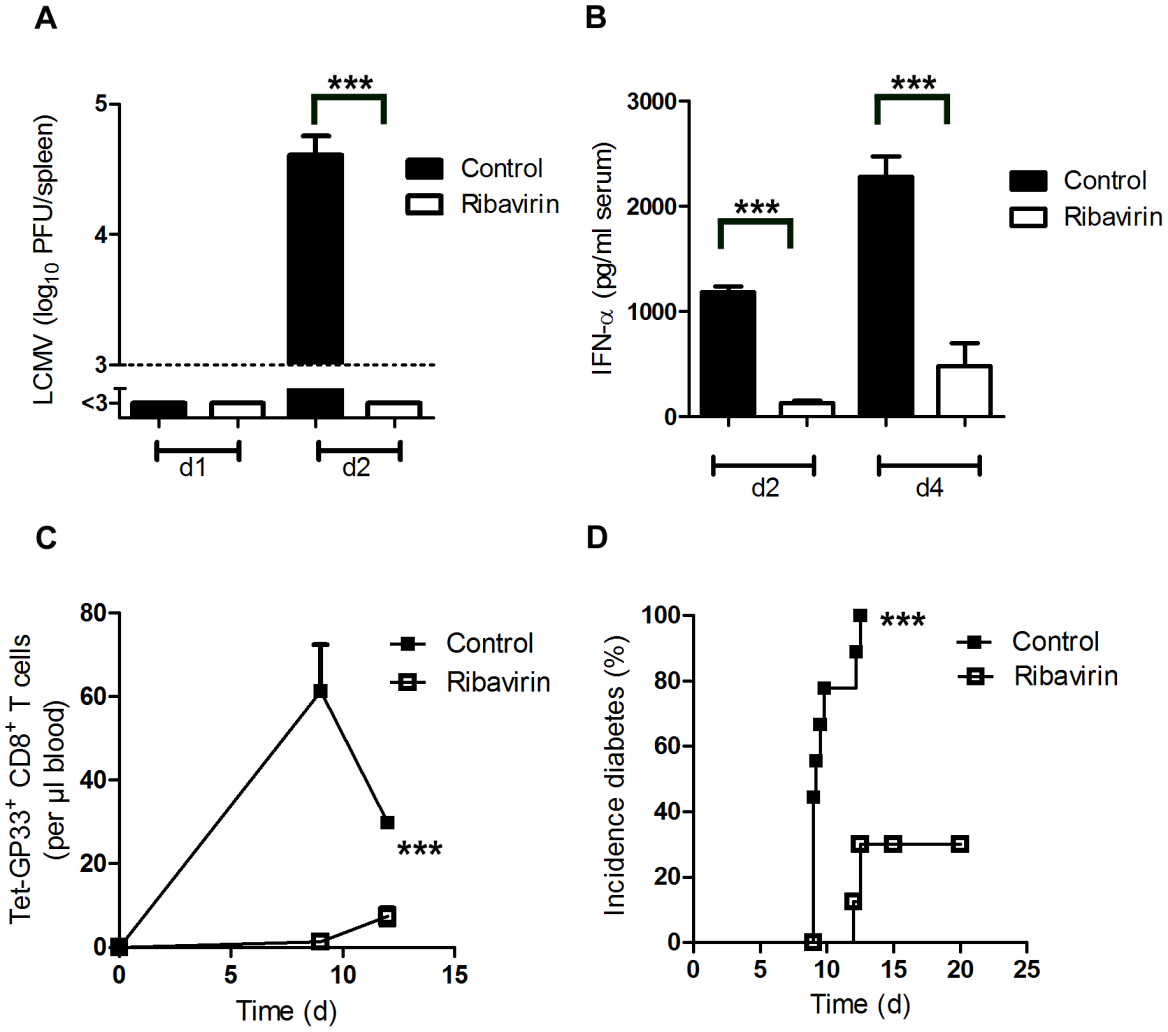
Pharmacologic inhibition of viral replication inhibits onset of autoimmune diabetes. (A–B) C57BL/6 mice were treated intraperitoneally with Ribavirin (5 mg daily), starting on day −3. On day 0, mice were infected with 200 PFU LCMV. LCMV titers in the spleen were measured by plaque assay on days 1 and 2 after infection (n = 5–7, A). Levels of IFN-α was measured in the serum by ELISA (n = 4–5, B). (C–D) RIP-GP mice were treated intraperitoneally with Ribavirin (5 mg daily), starting on day −3. On day 0, mice were infected with 200 PFU of LCMV-WE. Numbers of islet-specific CD8^+^ T cells were determined by tetramer staining and flow cytometry (C, n = 4). The onset of diabetes was assessed by measuring serum glucose concentrations at the indicated time points (D, n = 9). *** *P*<0.001 (Student's *t*-test) (A and B), two-way analysis of variance (ANOVA) (C) or Log-rank (Mantel-Cox) (D).

Besides enhancing early LCMV replication, dendritic cells are known to initiate the immune response by potent expression of co-stimulatory molecules (Figure S1). To see whether virus replication, and not other dendritic cell functions were essential to initiate autoimmune diabetes we treated mice with the anti-viral drug Ribavirin, which can efficiently suppress LCMV replication [31]. Indeed Ribavirin treatment was associated with significantly suppressed early LCMV replication (Figure 2A). In line with that innate antiviral IFN-α production was reduced in Ribavirin treated mice (Figure 2B). Reduced early virus replication blunted LCMV-specific CD8^+^ T cell priming and prevented onset of diabetes (Figure 2C and D).These data imply that indeed early virus replication in dendritic cells is essential to break immunological tolerance.

### Expression of *Usp18* in dendritic cells contributes to early virus replication and onset of diabetes

**Figure 3:**
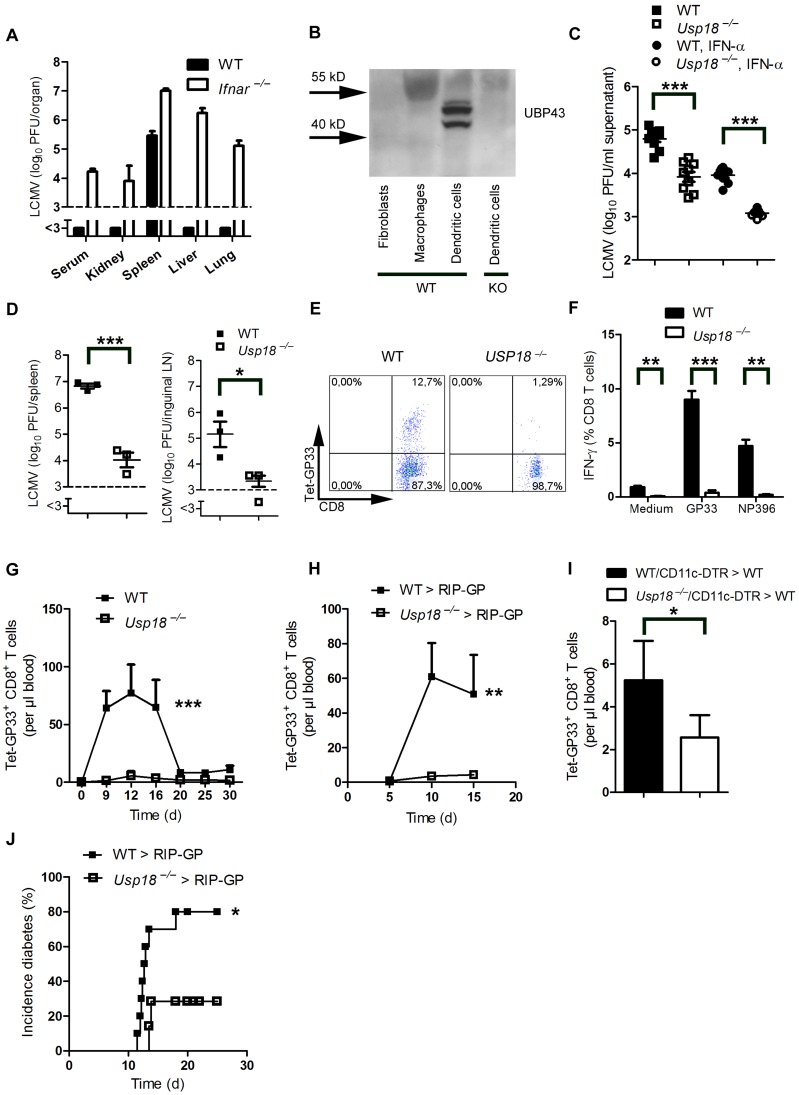
Expression of *Usp18* in dendritic cells guarantees early viral replication and onset of autoimmune diabetes. (A) WT and *Ifnar^−/−^* mice were infected with 200 PFU of LCMV-WE. Viral titers were analyzed in various organs by plaque assay on day 4 (n = 4). (B) Expression of UBP43 (protein encoded by *Usp18*) was assessed by Western blot in bone marrow–derived dendritic cells, macrophages, and fibroblasts (n = 3). Dendritic cells from *Usp18^−/−^* mice served as a control for antibody specificity. (C) Bone marrow–derived dendritic cells from WT or *Usp18^−/−^* mice were infected with LCMV *in vitro* (MOI = 1) or left uninfected. In addition, cells were treated with recombinant IFN-α (50 U/mL) or left untreated. After 48 hours, LCMV titers were measured in the culture supernatants by plaque assay (n = 9). (D) WT and *Usp18^−/−^* mice were infected with LCMV 2×10^6^ PFU. After one day viral titers were measured in the spleen and lymph nodes by plaque assay (n = 3). (E) FACS analysis of GP33^+^ CD8^+^ T cells measured in splenocytes from WT or *Usp18^−/−^* mice on day 7 after infection with 200 PFU LCMV-WE (One of three is shown). (F) FACS analysis of IFN-γ^+^ GP33^+^/and NP396^+^ CD8^+^ T cells measured in splenocytes from WT or *Usp18^−/−^* mice on day 7 after infection with 200 PFU LCMV-WE six hours after restimulation with GP33-peptide (n = 3–4). (G) FACS analysis of GP33^+^ CD8^+^ T cells measured in blood from WT or *Usp18^−/−^* mice at different time points after infection with 200 PFU LCMV-WE (n = 4–6, G). (H) RIP-GP mice were lethally irradiated and one day later were reconstituted with 10^7^ bone marrow from either *Usp18^−/−^* mice or WT littermate control mice. Thirty days later, mice were infected with 200 PFU LCMV. GP33-specific CD8^+^ T cells in the blood were counted by flow cytometry at the indicated time points after LCMV infection (n = 4). (I) C57BL/6 mice were lethally irradiated and one day later were reconstituted with a 1∶1 mixture of bone marrow derived from *Usp18^−/−^* and CD11c-DTR mice or from WT and CD11c-DTR mice as control animals. Thirty days later, mice were treated intraperitoneally with diphtheria toxin (10 µg/kg) on days −1, 2, 5, and 8 and were infected intravenously with 2×10^6^ PFU LCMV-WE on day 0 (n = 4). GP33-specific CD8^+^ T cells were assessed in peripheral blood 8 days after LCMV infection by tetramer staining and flow cytometric analysis. Results of 2 experiments are pooled. (J) RIP-GP mice were lethally irradiated and one day later were reconstituted with 10^7^ bone marrow cells from either *Usp18^−/−^* mice or WT littermate control mice and were infected with 200 PFU of LCMV 30 days later. The incidence of autoimmune diabetes was determined by measuring serum glucose concentrations at the indicated time points (n = 7–10). * *P*<0.05, ** *P*<0.01 and *** *P*<0.001 (Student's *t*-test) (C, D, F and I), two-way analysis of variance (ANOVA) (G and H) or Log-rank (Mantel-Cox) (J).

We found that LCMV replicated in dendritic cells in spleen and lymph nodes. In other organs no virus replication was detected, due to suppression of virus replication by IFN-I (Figure 3A). Therefore we wondered whether expression of endogenous inhibitors of IFN-I signaling in dendritic cells contributed to LCMV replication in dendritic cells. *Usp18* (UBP43) binds to the Jak1 binding site of the type I interferon receptor and inhibits its phosphorylation [32]. Therefore *Usp18* is a very efficient IFN-I inhibitor. First we analyzed expression of UBP43 in dendritic cells. Naïve dendritic cells, but not bone marrow–derived macrophages or fibroblasts, exhibited high expression of UBP43 (Figure 3B). Absence of UBP43 in *Usp18^−/−^* mice [33] reduced LCMV replication in DCs *in vitro* (Figure 3C) and was associated with reduced LCMV replication in spleen and lymph nodes *in vivo* (Figure 3D). These findings demonstrate that LCMV replication is enforced in dendritic cells as a consequence of *Usp18* expression. To test the role of *Usp18* in priming virus specific CD8^+^ T cells we infected WT and *Usp18^−/−^* mice with 200 PFU LCMV. The absence of *Usp18* strongly impaired expansion of antiviral CD8^+^ T-cells in the spleen till day 7 (Figure 3E). Reduced frequencies of virus specific CD8^+^ T cells were in line with reduced numbers of IFN-γ producing CD8^+^ T cells after restimulation (Figure 3F). In the blood *Usp18^−/−^* mice showed limited frequencies of virus-specific CD8^+^ T cells (Figure 3G). Although CD8^+^ T cells were reduced virus could be controlled in *Usp18^−/−^* mice (Figure S2). Next we generated bone marrow chimeras by transferring *Usp18^−/−^* bone marrow into irradiated RIP-GP mice to analyze the role of *Usp18* on virus induced autoreactive CD8^+^ T cells. Lack of *Usp18* on bone marrow derived cells blunted autoreactive CD8^+^ T cell response (Figure 3H). To underline the role of *Usp18* in CD11c expressing cells we generated mixed bone marrow chimeras by using *Usp18^−/−^* bone marrow mixed 1∶1 with bone marrow from CD11c-DTR mice in C57BL/6 wild-type mice. Diphtheria toxin treatment of these chimeric mice will deplete *Usp18*-competent DCs derived from CD11c-DTR mice but not *Usp18*-deficient DCs. Control mice were given a 1∶1 mixture of WT and CD11c-DTR bone marrow. Thirty days later, mice were treated with diphtheria toxin. After infecting these mice with LCMV we observed reduced expansion of islet-specific CD8^+^ T cells, implying that *Usp18* affects virus replication in DCs intrinsically (Figure 3I). Next we infected irradiated RIP-GP mice that had been reconstituted with bone marrow from *Usp18^−/−^* or WT mice with LCMV. The absence of autoreactive CD8^+^ T cells in *Usp18* deficient mice reduced the incidence of autoimmune diabetes, although mice still could control LCMV infection (Figure 3J). In conclusion, lack of *Usp18* in CD11c^+^ cells reduced priming of islet-specific CD8^+^ T cells and prevented induction of diabetes.

### Only replicating autoantigen is efficient in inducing autoimmune diabetes

**Figure 4:**
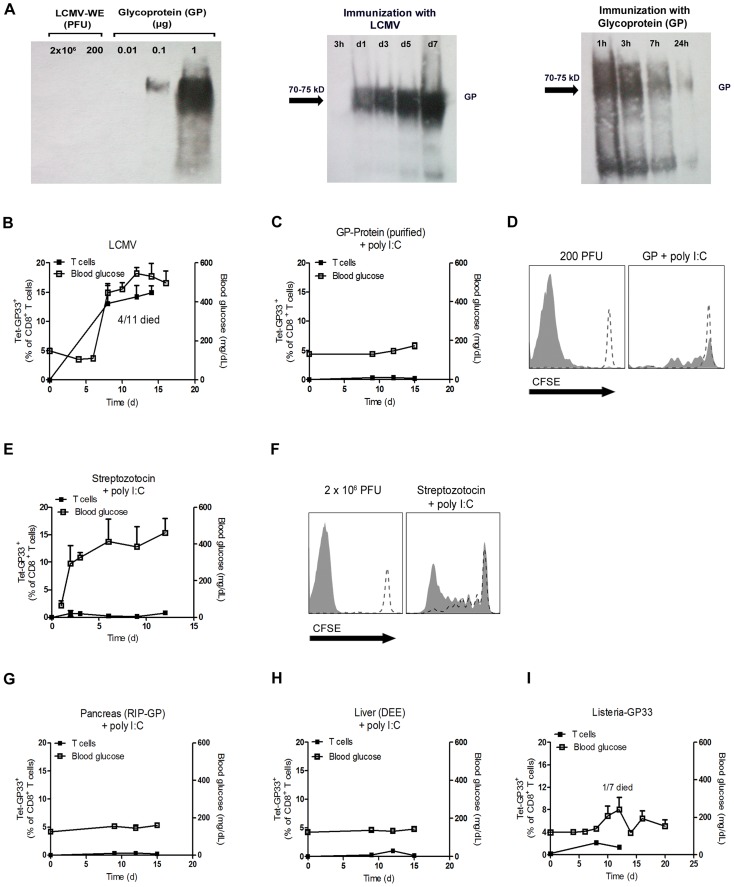
Only replicating antigen is efficient in breaking autoimmune tolerance. (A) Initial LCMV inoculate (2×10^6^ PFU and 200 PFU) and purified LCMV glycoprotein (GP) (0.01, 0.1, 1 µg) were stained for LCMV-GP by Western blot analysis. After immunization of C57BL/6 mice with live LCMV (200 PFU, i.v.) and purified LCMV glycoprotein (GP, 2 µg, i.v.), spleen lysates were analyzed by Western blot for LCMV-GP expression at the indicated time points. (B) RIP-GP mice were infected intravenously with 200 PFU of LCMV-WE. The number of GP33-specific CD8^+^ T cells was determined by tetramer staining and flow cytometry, and serum glucose concentration was determined at the indicated time points (n = 4–11). (C) RIP-GP mice were immunized intravenously with 2 µg purified LCMV-GP in combination with poly I:C (100 µg). The number of autoreactive CD8^+^ T cells was determined by tetramer staining and flow cytometry, and serum glucose concentration was determined at the indicated time points (n = 4). (D) 10^7^ Splenocytes from P14/CD45.1 mice were labeled with CFSE and adoptively transferred into C57BL/6 mice. After 24 hours, C57BL/6 mice were left uninfected (both histogram blots, dotted line) or infected with 200 PFU LCMV-WE (left histogram blot, filled area) or immunized with 2 µg LCMV-GP (n = 3, right histogram blot, filled area). Proliferation of CD45.1^+^CD8^+^ T cells was assessed by CFSE dilution in spleen 6 days after transfer. Histograms show cells gated on CD45.1^+^ CD8^+^ T cells. One representative set of data is shown. (E) RIP-GP mice were treated intraperitoneally with Streptozotocin (5 mg) and intravenously with poly I:C (100 µg). The number of islet-specific CD8^+^ T cells was determined by tetramer staining and flow cytometry, and serum glucose concentration was determined at the indicated time points (n = 3). (F) 10^7^ Splenocytes from P14/CD45.1 mice were labeled with CFSE and transferred into RIP-GP mice or C57BL/6 mice. After 24 hours, RIP-GP mice were treated either left untreated (right histogram blot, dotted line) or were treated intraperitoneally with 5 mg Streptozotocin and intravenously with poly I:C (100 µg, right histogram blot, filled area). C57BL/6 mice were either left untreated (left histogram blot, dotted line) or infected with 2×10^6^ PFU LCMV (left histogram blot, filled area). Proliferation of CD45.1^+^CD8^+^ T cells was assessed by CFSE dilution in the spleen 6 days after transfer (n = 3). Blots show cells gated on CD45.1^+^ CD8^+^ T cells. One representative set of data is shown. (G) RIP-GP mice were immunized intraperitoneally with homogenized pancreas (40 mg) from RIP-GP mice and intravenously with poly I:C (100 µg). The number of islet-specific CD8^+^ T cells was determined by tetramer staining and flow cytometry, and serum glucose concentrations were determined at the indicated time points (n = 4). (H) RIP-GP mice were immunized intraperitoneally with homogenized liver derived from DEE mice (100 mg) and immunized intravenously with poly I:C (100 µg). The number of islet-specific CD8^+^ T cells was determined by tetramer staining and flow cytometry, and serum glucose concentration was determined at the indicated time points (n = 4). (I) RIP-GP mice were infected with 10^3^ CFU of Listeria-GP33 intravenously. Number of islet-specific CD8^+^ T cells was determined by tetramer staining and flow cytometry, and serum glucose concentration was measured at the indicated time points (n = 4–7).

We speculated that infection with replicating virus might be associated with the production of much higher amounts of autoantigen than treatment with soluble autoantigen. Western blot analysis showed that the initial virus inoculate did not contain measurable LCMV-GP as assessed by western blotting while 0.1 µg purified glycoprotein (GP) was clearly detectable (Figure 4A). In contrast, LCMV-GP was detected in increasing amounts in spleen lysates for up to 7 days following LCMV infection (Figure 4A). In contrast, immunization with 2 µg soluble LCMV-GP was associated with detectable LCMV-GP in the spleen for only 24 hours (Figure 4A). This finding indicated that the amount of antigen expressed in the spleen correlates with active replication of LCMV. Infecting RIP-GP mice with LCMV led to expansion of LCMV-specific CD8^+^ T cells associated with induction of autoimmune diabetes to 100% of the mice, as demonstrated by elevated serum glucose concentrations (Figure 4B) [27]. To compare the immunogenicity of replicating virus with soluble antigen, we immunized RIP-GP mice with soluble LCMV-glycoprotein (LCMV-GP) together with the TLR ligand polyinosinic-polycytidylic acid (poly I:C) at concentrations known to induce potent innate immune responses [34]. In contrast to replicating virus, soluble LCMV-GP failed to induce measurable numbers of GP33-specific CD8^+^ T cells in peripheral blood, and diabetes was not induced (Figure 4C). Transferring CFSE-labeled LCMV-specific transgenic CD8^+^ T cells (derived from P14 mice [35]) into mice immunized with soluble LCMV-GP and poly I:C revealed that nonreplicating LCMV-GP induced detectable but limited CD8^+^ T cell proliferation *in vivo* (Figure 4D). To determine whether self-antigen released during the damage of beta islet cells in conjunction with poly I:C treatment could activate LCMV-specific CD8^+^ T cells, we treated RIP-GP mice with Streptozotocin, which is directly toxic to beta islet cells [36]. Streptozotocin treatment in combination with poly I:C as a innate immune activator induced diabetes in RIP-GP mice (Figure 4E). However, induction of diabetes was most likely due to the direct toxic effects of Streptozotocin, since GP33-specific CD8^+^ T cells were not detected in peripheral blood after Streptozotocin treatment (Figure 4E). To analyze the ability of the released LCMV-GP in this experimental setting to activate autoreactive CD8^+^ T cells, we transferred CFSE labeled GP33-specific CD8^+^ T cells into RIP-GP mice and then treated them with Streptozotocin plus poly I:C. CFSE-labeled LCMV-specific CD8^+^ T cells showed detectable but limited proliferation (Figure 4F). This finding suggested that even massive destruction of pancreatic islet cells was not sufficient to break the immunological tolerance of GP33-specific CD8^+^ T cells even in the presence of an inflammatory environment induced by poly I:C treatment. Next, we administered RIP-GP mouse–derived pancreatic homogenates to naïve RIP-GP mice, again in combination with poly I:C to stimulate innate immunity. This treatment led to very limited expansion of GP33-specific CD8^+^ T cells and was not associated with induction of autoimmune diabetes (Figure 4G). Similarly, administration of liver homogenates derived from DEE mice [37] which express the LCMV-GP under the actin promoter in combination with poly I:C to RIP-GP mice only led to limited expansion of LCMV-specific CD8^+^ T cells and did not induce autoimmune diabetes (Figure 4H). Autoimmune diabetes was also not induced in RIP-GP mice infected with listeria expressing the glycoprotein of LCMV (Listeria-GP) again correlated with limited expansion of LCMV-specific CD8^+^ T cells (Figure 4I). In summary, we found that non- or poorly-replicating antigen, even in combination with innate immune activation, is very inefficient in priming of autoantigen-specific CD8^+^ T cells. Only virus infection, supported by the *Usp18* driven enforced virus replication process in CD11c^+^ APCs is efficient in breaking immunologic tolerance to pancreatic islet cells in our model.

### Lack of early virus replication limits break of tolerance in RIP-NP diabetes model

**Figure 5:**
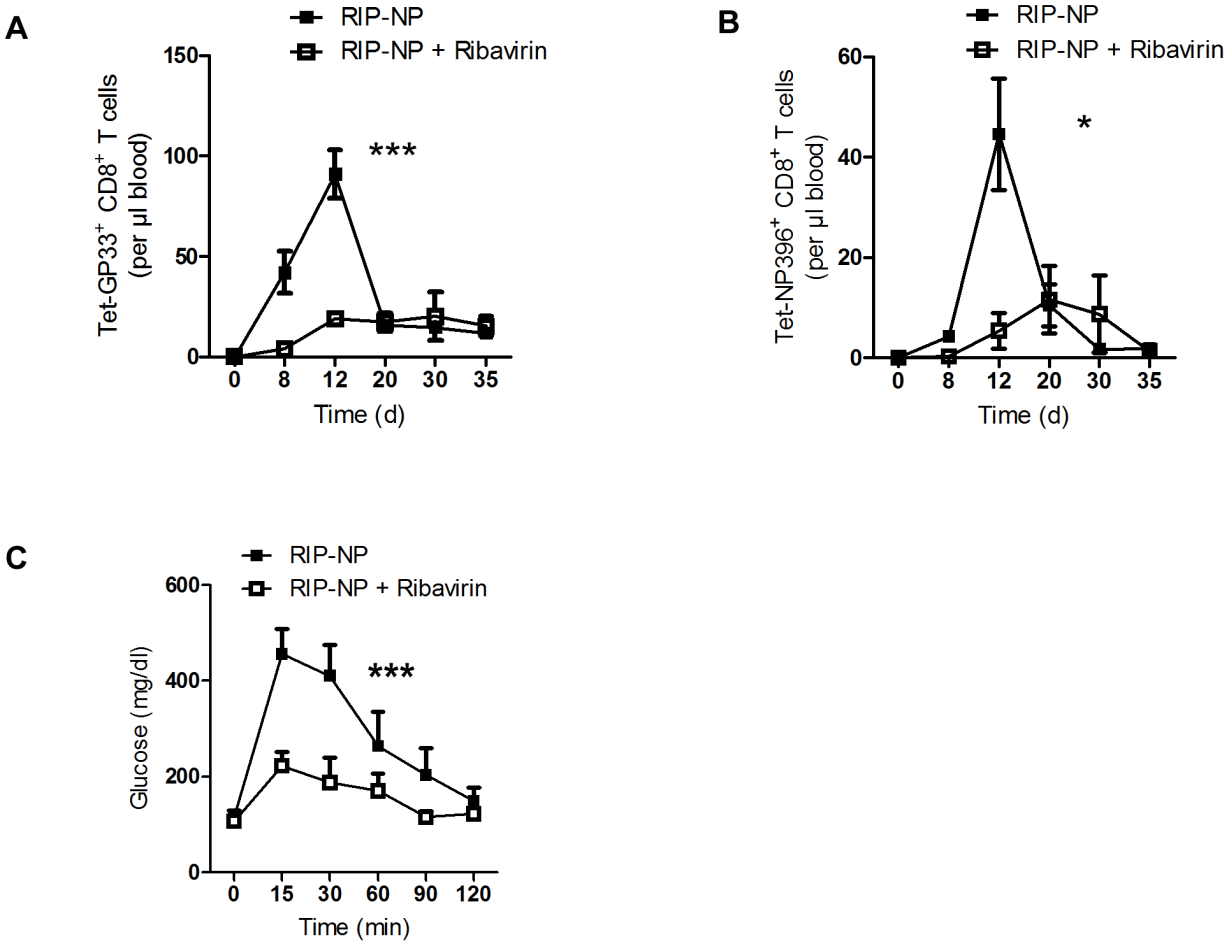
Ribavirin blunts auto-reactivity in RIP-NP diabetes model. RIP-NP mice were treated intraperitoneally with Ribavirin (5 mg daily), starting on day −3. On day 0, mice were infected with 200 PFU of LCMV-WE. (A) Numbers of virus-specific Tet-GP33^+^ CD8^+^ T cells were determined by tetramer staining and flow cytometry (n = 5–6). (B) Numbers of autoreactive Tet-NP396^+^ CD8^+^ T cells were determined by tetramer staining and flow cytometry (n = 5–6). (C) On day 50 glucose tolerance test was performed (n = 5–6). * *P*<0.05 and *** *P*<0.001 two-way analysis of variance (ANOVA).

The LCMV RIP-GP model is a model of acute onset of diabetes. The concurrent activation of the adaptive and innate immune response is essential to induce diabetes in this model [34]. In humans diabetes is often induced over a long period of time or in two or more events [38]. We found that enforced virus replication is activating both innate and adaptive immune response. Therefore it remains questionable if early virus replication can impact on diabetes in a model which is almost independent of innate immune activation. To get insights we infected RIP-NP mice with 200 PFU LCMV-WE. RIP-NP mice show partial expression of LCMV-NP in the thymus and therefore typically show a delayed onset of diabetes [39], [40]. Similar to our previous results, induction of antiviral LCMV-GP-specific CD8^+^ T cells was reduced by Ribavirin treatment in the RIP-NP mice (Figure 5A). Induction of autoreactive LCMV-NP-specific CD8^+^ T cells was in addition limited in the absence of enforced virus replication (Figure 5B). In line with these results mice treated with Ribavirin showed enhanced beta islet function compared to control mice (Figure 5C).

## Discussion

In this study we examined why replicating self-antigen is much more efficient in breaking autoimmune tolerance than the exposure to nonreplicating self-antigen. Our findings emphasize that the development of autoimmune diabetes requires active autoantigen replication in specialized APCs that are characterized by the expression of *Usp18*, a known inhibitor of type I interferon signaling [32]. Since *Usp18* expressing APCs are not responsive to the antiviral actions of type I interferons, they act as endogenous “replicators” of autoantigen.

Recently we found that expression of *Usp18* in CD169^+^ macrophages is important for initiating neutralizing antibodies against vesicular stomatitis virus [26]. In light of our data here we suggest that this mechanism is also of importance in dendritic cells for initiating innate and adaptive immune response against LCMV. In addition to LCMV replication, high expression of *Usp18* in DCs could explain the long-known phenomenon that DCs can be easily infected with several viruses [41]. Administration of autoantigen in various non-replicating forms only led to very limited activation and expansion of autoimmune CD8^+^ T cells, suggesting that the mechanism of enforced virus replication could be an essential factor allowing the immune system to differentiate between foreign and self-antigen.

In addition to (auto)antigen amplification, *Usp18* may have other functions in DCs. Indeed, lack of *Usp18* expression reduces the number of CD11b^+^ dendritic cells by 50% [42]. We found that after treatment with poly I:C, expression of MHC I and the costimulatory molecule CD80 was enhanced in *Usp18^−/−^* DCs compared to WT DCs (data not shown), implying that there is no general activation defect in *Usp18*-deficient DCs. In fact, the absence of IFN signaling (as in WT DCs), rather than enhanced interferon signaling (as in *Usp18^−/−^* DCs), impairs DC functions such as proteasomal degradation, cross-priming [43], [44], and costimulation [45]. Therefore, we hypothesize that lack of antigen amplification is the major defect in *Usp18^−/−^* DCs.

Immunohistological co-stainings revealed that LCMV replicates in the spleen mainly in CD169^+^ macrophages and CD11c^+^ cells (data not shown). Depletion of both cell types in CD11c-DTR mice completely blunted early LCMV replication in the spleen, while depletion of CD169^+^ macrophages in CD169-DTR mice showed no reduction in early LCMV titers (data not shown). This suggests that contribution of LCMV replication in CD169^+^ macrophages to total splenic LCMV replication is minor.

The results of several studies suggest that viral infection may be linked to the onset of human autoimmune diabetes. Using mouse models, we and others have demonstrated that this association can be explained by the activation of pattern recognition receptors during disease onset [34], [46]. Especially IFN-I enhances antigen presentation and induces an inflammatory status in beta islet cells [34]. Recent genetic analyses have indeed found that genes regulating the interferon response are important contributors to onset of diabetes [8], [9], [47], [48]. In particular, enhancement of the activity of the pattern recognition receptor RIG I is linked to a high risk of diabetes onset [47]. Therefore enhanced activity of *Usp18* in beta islet cells would limit IFN-I signaling in these cells and could prevent diabetes during exposure to IFN-I [49]. We found here that lack of *Usp18* in dendritic cells prevented enforced virus replication and would therefore limit induction of autoreactive CD8^+^ T cells, but also induction of IFN-I production. Therefore we would suggest that *Usp18* expression in dendritic cells could drive autoimmune diabetes by promoting activation of cross-reactive CD8^+^ T cells, but also by induction of high levels of IFN-I. Whether indeed the expression of interferon inhibitors such as *Usp18* in certain cell types contributes to the risk of human diabetes remains to be tested.

It still remains to be explained how bacteria, that express cross-reactive antigen might contribute to autoimmune diabetes induction. In humans there is no clear link between certain bacterial infection and onset of diabetes [18]. We found, using recombinant LCMV-GP33 expressing facultative intracellular *Listeria monocytogenes* that indeed low doses of systemic bacterial infection did not induce diabetes in RIP-GP mice. While this suggests that amplification of virus antigen was more efficient to break immunological tolerance, the contribution of intracellular bacterial amplification to the overall autoimmune activation remains to be studied.

We demonstrate here an *Usp18* driven mechanism which allows replicating virus, but not non-replicating autoantigen to break immunological tolerance. Blocking *Usp18* may be a potential target for pharmacological interference in the early pathogenic steps leading to the induction of autoimmune diabetes in humans.

## Methods

### Mice

All experiments were performed with the animals housed in single ventilated cages, with the authorization of Veterinäramt Nordrhein Westfalen (Düsseldorf, Germany) in accordance with the German law for animal protection or institutional guidelines of the Ontario Cancer Institute. Project was licensed under identification number (84-02.04.2011, A246). Rat insulin promoter-glycoprotein (RIP-GP) or promoter-nucleoprotein (RIP-NP) mice [27], [39], which express the LCMV glycoprotein or LCMV nucleoprotein respectively as a transgene under the rat insulin promoter, were used for the analysis of autoimmune diabetes and were maintained on a C57BL/6 background. P14 mice expressing a LCMV-GP33-41 specific TCR as a transgene were used for adoptive transfer experiments and were also maintained on a C57BL/6 background [35]. Mice expressing CD45.1 were used to track cells in adoptive transfer experiments. DEE mice express LCMV-GP under the actin promoter [37]. *Ifnar^−/−^* mice [34] and CD11c-DTR mice [28] were maintained on C57BL/6 background. *Usp18^−/−^* mice were generated in the Zhang lab and bred heterozygously on a Sv129×C57BL/6 background F4 and directly compared with littermate control animals.

### Lymphocyte transfer

Splenocytes from P14 mice expressing CD45.1 were labeled with carboxyfluorescein succinimidyl ester (CFSE, 1 µM, Invitrogen) and were injected intravenously into RIP-GP or C57BL/6 mice. One day later, mice were infected with LCMV-WE (200 PFU or 2×10^6^ PFU) or with purified LCMV glycoprotein or were treated with Streptozotocin (5 mg). Five days after LCMV infection, the proliferation of P14 T cells was assessed in the spleen by CFSE dilution and flow cytometry.

### Bone marrow chimeras

For the generation of bone marrow chimeras, recipient mice were irradiated with 9.5 Gy (320 kV X-rays, 3 Gy/min, 0.35 mm copper +1.5 mm aluminium filter; Pantak-Seifert, Ahrensburg, Germany) on day -1. On the next day, 10^7^ bone marrow cells were transferred. After 15 days Clodronate-Liposomes were administered to ensure macrophages exchange in *Usp18^−/−^*>RIP-GP, WT>RIP-GP, CD11c-DTR>RIP-GP, WT>RIP-GP, WT/CD11c-DTR>WT and *Usp18^−/−^*/CD11c-DTR>WT chimeras. Infections with LCMV were performed after 30 days.

### Cell culture, generation of murine primary cells

To generate primary macrophages, we isolated bone marrow from femurs and tibias of mice and eliminated erythrocytes. Bone marrow cells were cultured in very low endotoxin Dulbecco's Modified Eagle Medium (VLE-DMEM) supplemented with 10% (v/v) fetal calf serum (FCS) and 0.1% (v/v) ß-mercaptoethanol (ß-ME) and 20% (v/v) macrophage colony-stimulating factor (M-CSF). On day 9 or 10 of differentiation, cells were harvested for use in subsequent experiments. To generate primary fibroblasts, we removed the lungs of mice and digested them with DNase and Liberase for 60 min at 37°C. After being flushed through a strainer, cells were cultivated in DMEM supplemented with 10% (v/v) FCS and penicillin-streptomycin glutamine (PSG). On day 3, adherent cells were rinsed with fresh growth medium. After 3 more days of cultivation, differentiated fibroblasts were split. On day 10, fibroblasts were used for experiments. To generate conventional dendritic cells (cDCs) we isolated bone marrow taken from femurs and tibias of mice. Erythrocytes were eliminated. We cultured bone marrow cells in very low endotoxin Dulbecco's Modified Eagle Medium (VLE-DMEM) supplemented with 10% fetal calf serum (FCS) and 0.1% ß-mercaptoethanol (ß-ME) in the presence of granulocyte macrophage colony-stimulating factor (GM-CSF). On day 3 of differentiation, an equal volume of growth medium was added. Growth medium was exchanged on day 6 of differentiation. On day 9 or 10 of differentiation, cells were harvested for use in subsequent experiments.

### Virus and plaque assay

LCMV strain WE was originally obtained from F. Lehmann-Grube (Heinrich Pette Institute, Hamburg, Germany) and was propagated in L929 cells. Mice were infected intravenously with LCMV at the indicated doses. Viral titers were measured in a plaque-forming assay using MC57 cells as previously described [30].

### Bacteria

*Listeria monocytogenes* (L.m.) expressing the LCMV-GP33 as transgene was grown overnight in brain–heart infusion broth or thawed from frozen aliquots, washed two times in phosphate-buffered saline (PBS), and injected intravenously in 200 µl into the tail vein. 10^3^ CFU of *L.m*. intravenously was used as low-dose infection.

### Pharmaceutical compounds

Ribavirin (Essexpharma, Belgium) was administered intraperitoneally (5 mg daily) starting on day −3 before LCMV infection. Streptozotocin was administrated (5 mg) intraperitoneally once on day 0. Twelve hours later, 400 µL of glucose solution (20% in PBS) was injected intraperitoneally to prevent severe hypoglycemia. Diphtheria toxin was injected intraperitoneally at a dose of 30 µg/kg or 10 µg/kg as indicated. For immune activation, 100 µg poly (I:C) (Amersham) was given intravenously per mouse.

### Immunization with LCMV-GP

HEK-GP cells, which express the LCMV glycoprotein (GP), were cultured in 40 mL DMEM +10% FCS and Hygromycin B (300 µg/mL) in a 150-cm^2^ tissue culture flask. After approximately 80% of the cells were confluent, cells were washed twice with PBS and cultured in 8 mL DMEM with no supplements. After 48 hours the supernatant was harvested, and the LCMV-GP that was released by the cells into the supernatant was purified with sepharose PD-10 desalting columns (GE Healthcare). Liver tissue derived from DEE mice was smashed in 1 ml PBS using tissue lyser (Qiagen). Mice were immunized intraperitoneally with 100 mg in 200 µl PBS. Pancreas tissue derived from RIP-GP mice was smashed in 1 ml PBS using tissue lyser (Qiagen). Mice were immunized intraperitoneally with 40 mg in 200 µl PBS.

### Flow cytometry

Tetramers were provided by the National Institutes of Health (NIH) Tetramer Facility. 20 µl blood was stained with allophycocyanin (APC)-labeled GP33 MHC class I tetramers (GP33/H-2Db) for 15 minutes at 37°C. After incubation, the samples were stained with anti-CD8 peridinin-chlorophyll-protein-complex (PerCP; BD Biosciences, Franklin Lakes, NJ) for 30 minutes at 4°C. Erythrocytes were then lysed using 1 ml BD lysing solution (BD Biosciences); washed 1× and analyzed with flow cytometer. Absolut numbers of GP33-specific CD8^+^ T cells/µl blood were calculated from FACS analysis using fluorescing beads (BD Biosciences).

### Blood glucose measurement, glucose tolerance test

Serum glucose concentrations were measured with a contour meter (Bayer, Leverkusen). Mice were considered diabetic if the glucose concentration was higher than 200 mg/dl. For glucose tolerance test, mice were fasted for 15 hours and then received a single intraperitoneally injection of 2 mg/g body weight glucose (Merck). Blood glucose was measured immediately before injection and then at 15, 30, 60, 90 and 120 minutes after injection.

### ELISA

IFN-α ELISA was performed according to the protocol of the manufacturers (PBL Interferon source).

### Histology

Conventional histology was performed as previously described [50]. Briefly, snap-frozen tissue was stained with rat anti-mouse polyclonal antibody to LCMV nucleoprotein (VL4; made in-house). Polyclonal anti-rat biotin antibody (eBioscience) and anti-biotin streptavidin peroxidase (Thermo Scientific) were then used before visualization with a 2-solution DAB staining kit (Invitrogen).

### Western blot

Proteins were isolated with trizol and solubilised with 10 M urea/50 mM DTT. Protein lysates were normalized for total protein (Bio-Rad). Proteins were analyzed by electrophoresis under denaturating conditions using 4–20% SDS ClearPAGE and blotted onto nitrocellulose membranes (Whatman). LCMV-GP was stained with KL25 antibody (made in-house) or UBP43 (Santa Cruz 98431)

### Statistical analysis

If not differently stated data are expressed as means and S.E.M. Student's *t*-test was used to detect statistically significant differences between groups, or Log-rank (Mantel-Cox) test to detect statistically significant differences of incidence of diabetes. Significant differences between several groups were detected by two-way analysis of variance (ANOVA). The level of statistical significance was set at *P*<0.05.

## Supporting Information

Figure S1 **Dendritic cells are activated during LCMV infection.** C57/BL6 mice were infected with 2×10^6^ PFU of LCMV. Expression of MHC-I and CD86 on splenic dendritic cells was analyzed at the indicated time points. Dotted line indicates staining with isotype antibody.
(TIF)

Figure S2 ***Usp18^−/−^*** **mice can cope with LCMV infection.** Virus titers in the spleen of WT or *Usp18^−/−^* mice measured on day 5 and 30 after infection with 200 PFU LCMV-WE using in plaque assay (n = 4) *** *P*<0.001 (Student's *t*-test).
(TIF)
