# Supplementary figures and images for: Usp18 Driven Enforced Viral Replication in Dendritic Cells Contributes to Break of Immunological Tolerance in Autoimmune Diabetes
Source: PLoS Pathog. 2013 Oct 24;9(10):e1003650. doi: 10.1371/journal.ppat.1003650 (PMC3812017; doi:10.1371/journal.ppat.1003650)

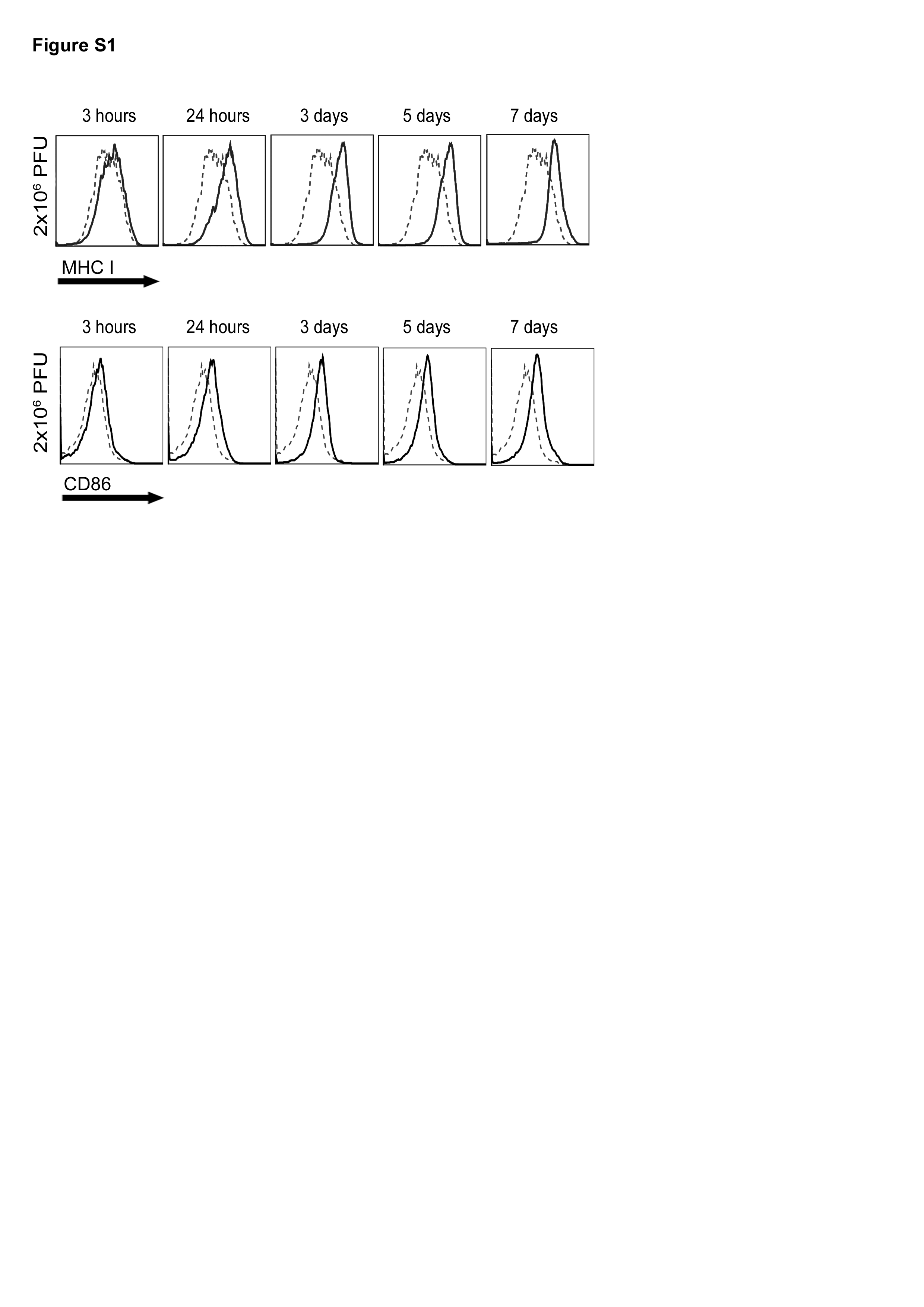

Supplement: Figure S1 — Dendritic cells are activated during LCMV infection. C57/BL6 mice were infected with 2×10^6^ PFU of LCMV. Expression of MHC-I and CD86 on splenic dendritic cells was analyzed at the indicated time points. Dotted line indicates staining with isotype antibody. (TIF) [file ppat.1003650.s001.tif]

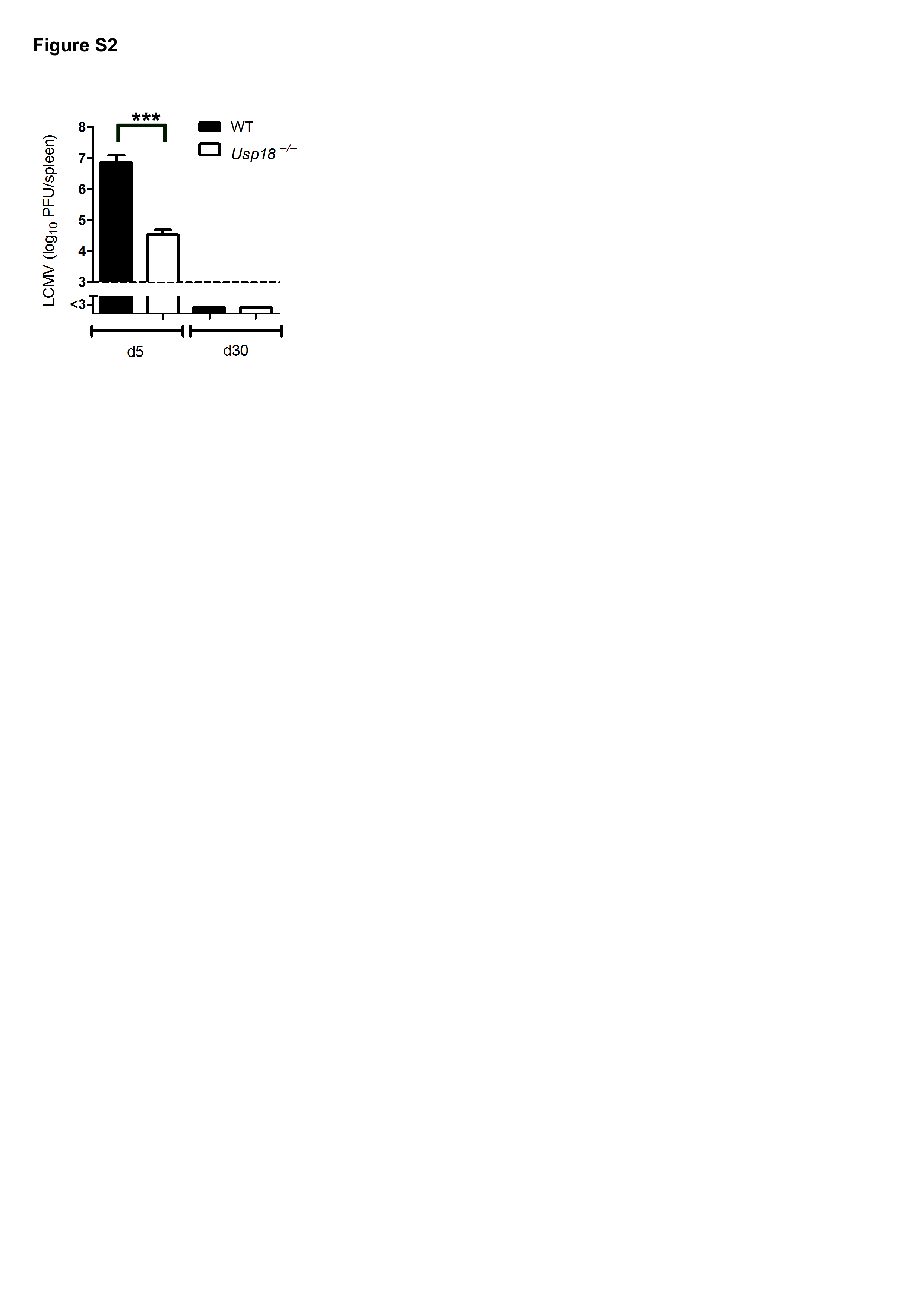

Supplement: Figure S2 — Usp18−/− mice can cope with LCMV infection. Virus titers in the spleen of WT or Usp18−/− mice measured on day 5 and 30 after infection with 200 PFU LCMV-WE using in plaque assay (n = 4) *** P<0.001 (Student's t-test). (TIF) [file ppat.1003650.s002.tif]
